# Artificial Intelligence-Based Quality Control of Pharmaceutical Tablets: A Visual Dataset and Benchmark for Automated Defect Detection

**DOI:** 10.3390/pharmaceutics18101287

**Published:** 2026-10-10

**Authors:** Tahir Mahmood, Sabi Ur Rehman, Arslan Niamat, Sung Jae Lee, Kang Ryoung Park

**Affiliations:** 1 Division of Electronics and Electrical Engineering, Dongguk University, 30 Pildong-ro 1-gil, Jung-gu, Seoul 04620, Republic of Korea; tahirmahmood@dongguk.edu (T.M.); sungje0319@dgu.ac.kr (S.J.L.); 2 Department of Pharmacy, Faculty of Natural Sciences, Forman Christian College (A Chartered University), Lahore 54600, Pakistan; sabirehman@fccollege.edu.pk (S.U.R.); arslanraja3107@gmail.com (A.N.)

**Keywords:** pharmaceutical tablet inspection, normal-versus-defective classification, deep learning, computer vision, image classification, pharmaceutical quality control

## Abstract

**Background/Objectives**: Automated visual inspection of pharmaceutical tablets is increasingly important for detecting manufacturing defects and maintaining consistent product quality. Progress in artificial intelligence-based defect classification is, however, constrained by the scarcity of publicly accessible image datasets covering defective tablets under diverse visual conditions. This study introduces the PharmaVision (PV) dataset, a public visual dataset and benchmark for classifying normal and defective compressed tablets. **Methods**: The PV dataset contains 640 smartphone-acquired images spanning varied tablet products, backgrounds, and lighting, with fixed tablet-level splits (190/190 training, 30/30 validation, and 100/100 test images for the normal and defective classes, respectively). Twelve representative architectures spanning classical convolutional networks, mobile networks, and vision transformers were benchmarked under an identical protocol using accuracy, precision, recall, and harmonic mean of precision and recall (F1-score), with 95% confidence intervals and paired statistical tests. Cross-dataset generalization was assessed on the independent pillQC dataset without fine-tuning, and efficiency was profiled using parameters, Giga floating-point operations (GFLOPs), latency, and throughput. **Results:** EfficientNetV2-S achieved the highest F1-score on the PV test set (88.56%), although paired statistical tests did not distinguish it from Xception (87.08%) or Swin-B (85.71%). On pillQC, GoogLeNet and EfficientNetV2-S showed the strongest cross-dataset performance, whereas both vision transformers declined relative to their in-domain results. AlexNet gave the highest theoretical throughput (602.41 frames per second (FPS), median latency 1.66 ms), whereas ShuffleNetV2 and MobileNetV3-small offered the smallest parameter counts. **Conclusions**: In-domain accuracy, cross-dataset robustness, and measured latency diverged, with no single architecture best on all three axes. The PV dataset, code, and evaluation protocol are publicly available on GitHub to support reproducible AI-based tablet quality control.

## 1. Introduction

The pharmaceutical industry continuously adopts advanced manufacturing technologies and quality control strategies to ensure the production of safe, effective, and consistent medicines. Visual examination of solid oral dosage forms is an established element of good manufacturing practice, and the appearance of a tablet is a specified quality attribute rather than a cosmetic concern: a visible abnormality is the outward signature of an underlying process deviation and may accompany variation in dose integrity. Tablets can develop a wide range of visual and structural defects during manufacturing, handling, and packaging, including chipping, cracking, capping, lamination, mottling, discoloration, black or foreign specks, incomplete or incorrect printing, missing or unclear imprints, uneven coating, coating peeling, surface roughness, deformation, broken edges, and irregular size or shape [1,2]. These arise from variations in raw material and granule properties, powder flowability, compression conditions, tooling characteristics, coating-process parameters, environmental conditions, and mechanical stresses encountered during manufacturing and subsequent handling [3]. Reliable identification of such abnormalities before medicines reach patients is therefore an essential component of pharmaceutical quality assurance.

In pharmaceutical manufacturing facilities, the inspection of tablets continues to depend on manual visual examination, which is time-consuming, labor-intensive, subjective, and vulnerable to human fatigue, particularly when thousands of tablets must be examined within a limited period. Inspectors may differ in their interpretation of acceptable and defective tablets, and small or subtle defects may be overlooked. The scale of modern continuous manufacturing has motivated interest in 100% in-line visual inspection of tablets produced by direct compression and coating [4], a volume at which manual examination is no longer practical. Rule-based machine vision systems were introduced to reduce this variability: early work addressed real-time tablet segmentation [5] and the automated assessment of imprint quality [6]. These systems perform well under the controlled illumination and fixed geometry for which they are configured, but their reliance on hand-tuned features and thresholds limits their ability to generalize across different tablet shapes, colors, imprints, orientations, imaging backgrounds, and defect appearances.

Deep learning has become a promising alternative because it learns discriminative image features directly from data rather than from hand-crafted rules [7]. Its effectiveness is well established in industrial surface-defect inspection, where public benchmarks have made competing methods directly comparable, and in adjacent visual inspection domains such as medical image analysis [8]. Several studies have applied deep learning specifically to pharmaceutical visual inspection [9,10,11,12,13]. Quan et al. used images from a multi-camera pharmaceutical inspection system to train a deep learning classifier that distinguished good from defective tablets by transfer learning, demonstrating feasibility on industrially acquired imagery that was not released as an open benchmark [14]. Rački et al. proposed a compact convolutional neural network (CNN)-based architecture for the segmentation and scoring of surface defects on solid oral dosage forms, evaluated on challenging real-world inspection data and shown to support real-time operation [15]. Ficzere et al. combined machine vision with deep learning for simultaneous coating thickness measurement and defect recognition on film-coated tablets [16]. Across these studies, deep architecture consistently outperforms earlier hand-crafted approaches, but each was developed and validated on a distinct, unreleased dataset with its own imaging setup and defect taxonomy, so none of the reported accuracy can be independently verified or compared on common ground.

Despite these advances, public and reproducible datasets for normal-versus-defective classification of compressed pharmaceutical tablets remain scarce. One such public resource, pillQC provides labeled images of normal tablets and tablets with chips and dirt. However, the dataset was acquired for a single tablet product under a fixed and controlled imaging configuration. It therefore cannot be used to assess whether a learned representation generalizes across tablet shapes, colors, imprints, backgrounds, illumination conditions, or capture devices, precisely the variability an inspection system encounters in practice. No public resource currently supports training and comparison of compressed-tablet defect classifiers across such diverse acquisition conditions under a fixed, published protocol.

This study addresses that gap by developing and benchmarking the foundation of an artificial intelligence-based inspection system for compressed pharmaceutical tablets: a system that analyzes a photograph of an individual tablet, cropped to the tablet region, and determines whether it is visually normal or carries a visible physical defect. Such a system is intended to support, rather than replace, the judgment of qualified quality-assurance and quality control personnel. To this end, we introduce the PharmaVision (PV) Dataset, a publicly available visual dataset for binary classification of normal and defective compressed tablets. The PV dataset combines tablets photographed during real pharmaceutical production with additional defective tablets prepared under controlled conditions by pharmacists, in order to broaden coverage of less frequently occurring defect types; labels reflect visual condition only and do not certify chemical, dissolution, or microbiological attributes. We benchmark the dataset using twelve deep learning architectures to establish a baseline for automated normal-versus-defective tablet classification. Beyond in-domain evaluation on the PV test set, models trained exclusively on the PV dataset are evaluated, without any adaptation or fine-tuning, on the independent pillQC dataset to assess cross-dataset generalization. Each model is additionally profiled in terms of trainable parameters, Giga floating-point operations (GFLOPs), inference latency, and throughput. The main contributions of this work are as follows:

- We propose a new publicly available dataset, the PV dataset, comprising 640 images of compressed pharmaceutical tablets, with fixed training (190/190), validation (30/30), and test (100/100) splits for the normal and defective classes, respectively. Images were partitioned at the level of the source tablet, verified by image matching so that no physical tablet appears in more than one split, and the three partitions were acquired in separate sessions approximately two weeks apart. The composition of the dataset is documented in terms of product types, coating, shape, color, and defect types.
- The dataset is benchmarked on twelve architectures spanning classical CNNs, mobile-oriented networks, and vision transformers, trained and evaluated under an identical protocol with no per-architecture tuning, providing future tablet inspection methods with a common reference point for comparison.
- To benchmark cross-dataset generalization, each model trained on the PV dataset is evaluated, without adaptation, on the independent pillQC dataset, quantifying how well its learned representation transfers to tablets, imaging conditions, and defect types outside its source distribution.
- Parameter count, GFLOPs, latency, and throughput are reported alongside accuracy, precision, recall, and harmonic mean of precision and recall (F1-score) for every architecture, allowing predictive performance to be weighed against the requirements of real-time, in-line inspection. In addition, the dataset, the augmented training set, and the complete training and evaluation code are released publicly [17], allowing independent retraining and verification of the reported results under the same protocol.

The remainder of this paper is organized as follows: Section 2 describes construction of the PV dataset and the experimental protocol. Section 3 reports classification results on the PV test set, cross-dataset generalization to pillQC, statistical analysis of these results, and computational efficiency. Section 4 discusses the implications and limitations of these findings, and Section 5 concludes the paper.

## 2. Materials and Methods

### 2.1. PharmaVision: Tablet Quality Assessment Dataset

**Figure 1:**
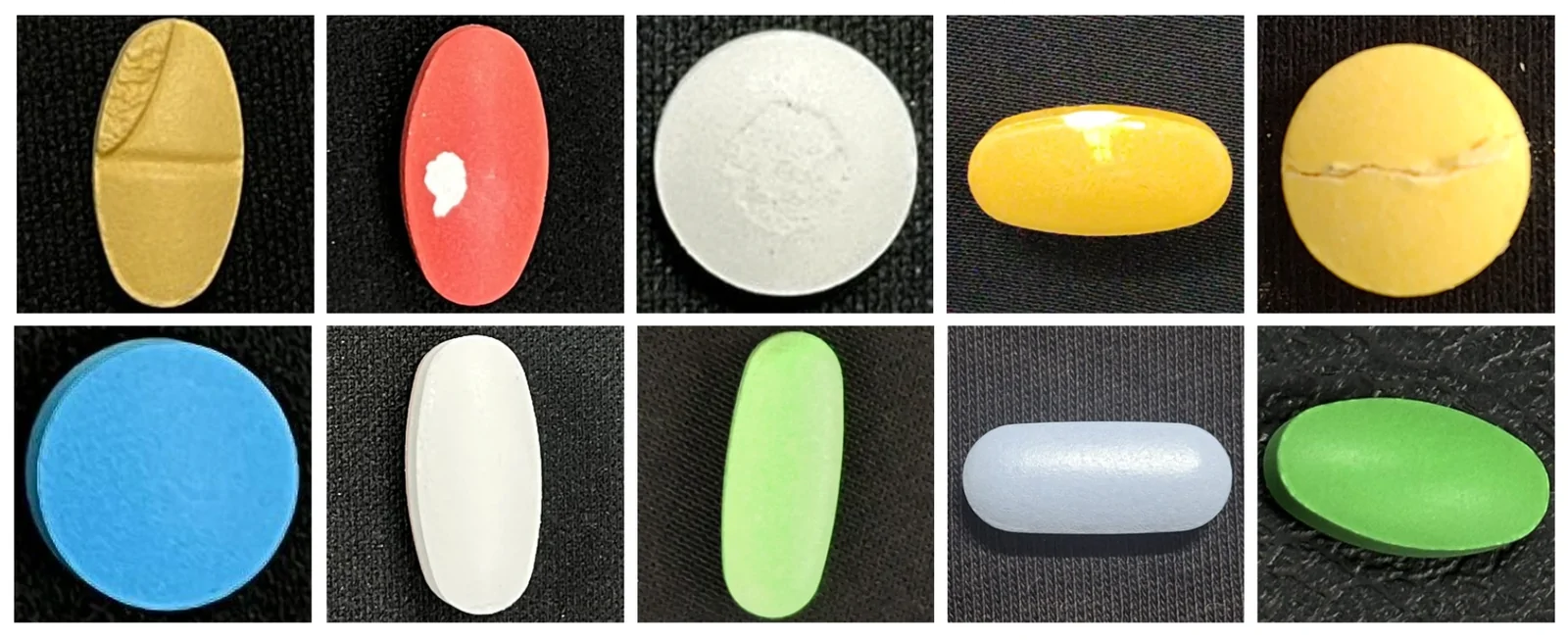
Representative samples from the proposed dataset, with defective tablet images shown in the top row and normal tablet images shown in the bottom row.

**Figure 2:**
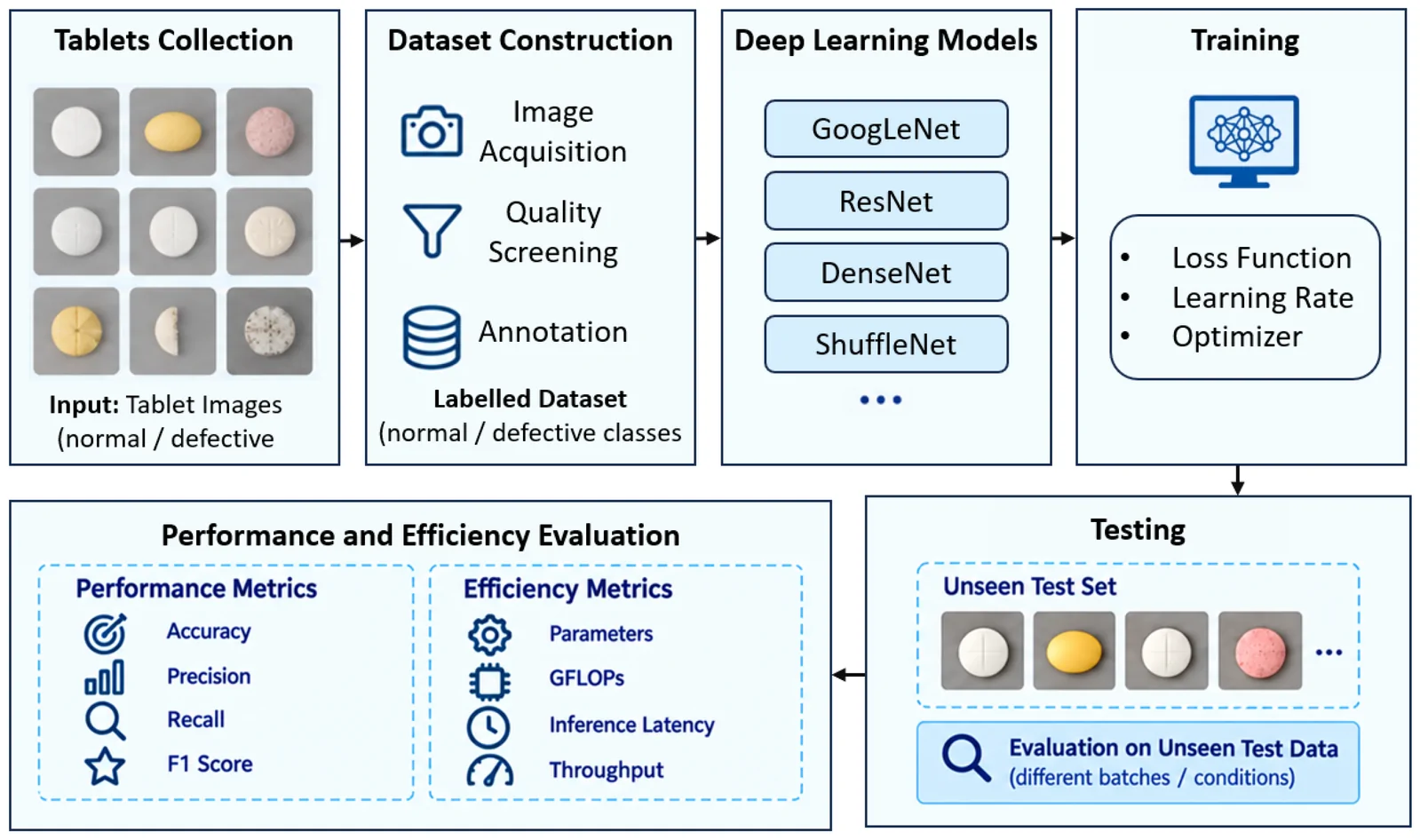
Overview of the proposed dataset development and benchmarking framework for automated normal-versus-defective classification of pharmaceutical tablets.

This study introduces the PharmaVision (PV) dataset, a visual dataset developed for artificial intelligence-based quality control of pharmaceutical tablets. The dataset is designed to support automated visual inspection of individual compressed tablets using deep learning methods, and to provide a fixed, reproducible benchmark for normal-versus-defective classification. In this work, the defective class was treated as the positive class for the calculation of precision, recall, and F1-score. The dataset contains 640 images, equally divided between the normal and defective classes, organized into predefined training, validation, and held-out test subsets. Representative examples of normal and defective tablets are presented in Figure 1, and the complete dataset-development workflow is illustrated in Figure 2.

#### 2.1.1. Dataset Development and Image Acquisition

The PV dataset combines two complementary sources of tablet images. A portion was collected directly from real pharmaceutical tablet manufacturing, capturing naturally occurring production defects together with normal tablets from the same batches. To broaden coverage of defect types and increase the number of defective samples available for training and evaluation, additional defective tablets were prepared under controlled conditions, in which pharmacists manually introduced physical defects onto normal tablets. This combined approach was intended to preserve the visual realism of genuine production defects while ensuring sufficient representation of less frequently occurring defect types.

All images were captured using consumer mobile phone cameras, specifically the Samsung Galaxy A35 [18] and Samsung Galaxy A05 [19], rather than specialized industrial imaging equipment. This choice reflects imaging conditions that are readily achievable in practice and demonstrates that meaningful tablet inspection is feasible without dedicated machine vision hardware. Images were captured at an approximate camera-to-tablet distance of around 30 cm, without a fixed jig or mount, so that minor variation in framing and distance is present across the dataset. Both devices were used across all three partitions, so that no split is associated with a single camera model. Each tablet was photographed individually against varying backgrounds and under varying lighting conditions, deliberately reflecting the range of visual conditions encountered in real manufacturing and inspection environments rather than a single fixed acquisition setup.

Following acquisition, each image was manually cropped to a region centered on the tablet, such that the tablet occupied most of the frame and extraneous background was reduced. All cropping was performed by the same operator using a consistent procedure applied identically across all partitions and both classes. Cropping was not blinded to class, because the class of each tablet was known at acquisition, but the crop was based solely on the tablet’s position in the frame and was not influenced by defect appearance. About 80% of crops were square; the remainder, of elongated tablets, were rectangular (Table 1). All images were resized to 224 × 224 pixels for training and evaluation (Section 2.3).

#### 2.1.2. Tablet Types and Defect Categories

Each image in the PV dataset was assigned one of two labels. Normal denotes a tablet with no visually observable defect. Defective denotes a tablet exhibiting one or more visually observable physical, structural, surface, coating, or appearance-related abnormalities. This binary framing was chosen because the dataset’s primary purpose is to support automated screening of tablets into acceptable and non-acceptable categories, the initial decision point in a practical quality control workflow, after which tablets flagged as defective would be routed for further review.

The dataset contains at most 550 unique physical tablets from 91 visually distinct product groups (Table 1); some tablets were photographed more than once, so the number of unique tablets is lower than the number of images. Product groups were defined from tablet appearance (imprint, color, shape and size); groups without a legible imprint may combine visually indistinguishable products. The products are commercially branded medicines, so manufacturer identities are not disclosed. Batch numbers, formulations, strengths and manufacturing processes were not recorded. Round (242 images), oval (89) and caplet or capsule-shaped (304) tablets are represented, together with four hard capsules and one heart-shaped tablet, in colors ranging from white and cream (247 images) to yellow, orange, red, brown, gray, blue and green. About two thirds of the images show film-coated tablets and one-third uncoated tablets, and many carry score lines, embossed logos or alphanumeric imprints. This variation, present in both the naturally sourced and manually prepared portions of the dataset, was intentional, so that models trained on the PV dataset learn to distinguish defect status from natural differences in tablet appearance rather than from a single fixed tablet type.

**Table 1:** Composition of the PV dataset by partition and class (N: normal; D: defective). Values are numbers of images, except for unique physical tablets and product groups. Unique physical tablets are upper bounds: images of the same tablet were identified by image matching with visual confirmation and counted once. Product groups were defined from tablet appearance (imprint, color, shape, and size); because a product can occur in several columns, product-group counts are not additive across columns, and the Total column gives the number of distinct groups. Coating was assessed visually.

Characteristic	Train N	Train D	Val N	Val D	Test N	Test D	Total
Images	190	190	30	30	100	100	640
Unique physical tablets	≤158	≤140	≤28	≤29	≤96	≤99	≤550
Product groups	35	30	7	5	44	32	91
Coating: film-coated	111	144	24	25	48	65	417
Coating: uncoated	76	46	4	5	51	35	217
Coating: sugar-coated	0	0	2	0	0	0	2
Coating: hard capsule	3	0	0	0	1	0	4
Shape: round	103	48	6	5	47	33	242
Shape: oval	12	17	12	18	14	16	89
Shape: caplet/capsule-shaped	72	125	12	7	38	50	304
Shape: other (hard capsule, heart-shaped)	3	0	0	0	1	1	5
Color: white/cream	78	75	4	5	43	42	247
Color: yellow	22	17	0	0	16	23	78
Color: orange/peach	23	34	0	0	17	21	95
Color: red/pink/maroon	9	4	1	0	11	5	30
Color: brown/olive	22	3	0	0	4	9	38
Color: gray/beige	8	16	0	0	0	0	24
Color: blue	23	41	11	18	6	0	99
Color: green/teal	3	0	14	7	3	0	27
Color: two-tone (capsule)	2	0	0	0	0	0	2
Imprint: none visible	115	98	24	16	39	50	342
Imprint: score line only	8	7	1	2	13	19	50
Imprint: text or logo	67	85	5	12	48	31	248
Non-square crop	43	53	4	6	9	16	131

The defective class includes chips (111 images), cracks (59), broken tablets with missing fragments (25), surface abrasion or erosion of the coating or imprint (75), discoloration, mottling, specks or stains (27), lamination (3), and combinations of these (20) (Table 2). Severity was graded as mild (a small edge chip, hairline crack or small spot, with the tablet outline intact and less than about 5% of the visible surface affected), moderate (a clearly visible chip, crack, abrasion or discoloration that alters the outline or affects about 5–20% of the surface) or severe (a fracture through the tablet, a missing fragment, or more than about 20% of the surface affected). Of the 320 defective images, 92 (28.8%) show naturally occurring production defects and 228 (71.3%) defects introduced manually by pharmacists. These defects appear on both plain, uncoated tablets and on coated, imprinted tablets, so that a model must learn to recognize defect status independently of tablet color, shape, or imprint pattern. Not every defective tablet exhibits the same type or severity of defect: some images contain a single obvious abnormality, such as a broken edge, while others exhibit multiple visible defects simultaneously. The defective category therefore represents a broad visual quality-failure class rather than a single defect mechanism.

In the present release, all such abnormalities are consolidated into a single defective label for binary classification. It should be recognized that this label reflects visual quality assessment only: a tablet labeled as normal is visually normal in the acquired image, and this label does not constitute confirmation of chemical composition, dosage uniformity, dissolution performance, microbiological quality, or any other non-visual pharmaceutical attribute. The dataset is accordingly intended as a visual defect-classification resource rather than a complete pharmaceutical-quality certification dataset. The per-defect-type distinctions recorded during acquisition are released alongside the binary labels and may support future extensions toward multi-class defect recognition, defect localization, or segmentation.

**Table 2:** Defect characteristics of the defective class by partition: primary defect type, severity, and origin (naturally occurring in production versus manually introduced by pharmacists). Severity criteria are defined in Section 2.1.2.

Defect Characteristic	Train (*n* = 190)	Val (*n* = 30)	Test (*n* = 100)	Total (*n* = 320)
Chip (edge or surface material missing)	67	18	26	111
Crack (fracture line, no fragment lost)	35	4	20	59
Broken (fragment missing or tablet split)	12	3	10	25
Abrasion (surface erosion, or imprint)	48	4	23	75
Discoloration, mottling, specks or stains	16	0	11	27
Lamination/layer separation	2	0	1	3
Multiple defect types	10	1	9	20
Severity 1—mild	43	13	19	75
Severity 2—moderate	115	13	64	192
Severity 3—severe	32	4	17	53
Origin: naturally occurring	44	3	45	92 (28.8%)
Origin: manually introduced	146	27	55	228 (71.3%)

#### 2.1.3. Image Diversity and Acquisition Conditions

To approximate the range of conditions an inspection system would encounter in practice, images were captured against multiple backgrounds and under multiple lighting conditions rather than a single controlled setup. This variation was introduced deliberately, for both the naturally sourced production images and the manually prepared defective images, to support generalization beyond a specific imaging environment and to reduce the risk of models learning background- or lighting-specific shortcuts rather than defect-relevant features. Backgrounds include a plain dark background, a wood-grain surface, and several solid-colored backdrops (including purple, teal, and green), photographed under the corresponding ambient lighting of each setting rather than a single studio configuration. Backgrounds and devices were distributed across both classes rather than being associated with a single label. The training, validation, and test partitions were acquired in separate sessions approximately two weeks apart. The leakage-control consequences of this design are described in Section 2.1.5.

#### 2.1.4. Dataset Annotation and Quality Control

Labeling proceeded in four stages: initial labeling at acquisition, independent blind review, curation and quality screening, and a final confirmation pass. In the first stage, the pharmacist who photographed each tablet assigned a normal or defective label based on the tablet’s visible condition at the time of capture. For the manually prepared defective tablets, this label records the defect deliberately introduced during preparation and is therefore not an independent visual judgment; for production-sourced tablets, it reflects visual assessment of the tablet in hand. In the second stage, a second pharmacist, who had not been present at acquisition and was not informed of the first label or of whether a tablet came from production or from controlled preparation, independently assigned a normal or defective label from the image alone. Disagreements were resolved through mutual discussion between the two pharmacists until consensus was reached; pre-consensus individual labels were not retained as separate records. In the third stage, two artificial intelligence (AI) researchers curated, organized the compiled dataset, and screened image quality prior to release. Images with uncertain, ambiguous, or otherwise unclear visual characteristics, including out-of-focus captures, insufficient tablet visibility, and borderline cases that could not be confidently assigned to either class, were excluded from the benchmark or returned for additional review. After curation, all images were compared pairwise within product groups using SIFT key points matching [20] with geometric verification, followed by visual confirmation, to detect the same physical tablet in more than one partition; no physical tablet appears in more than one partition of the released dataset. In the fourth stage, the compiled dataset was returned to the pharmacists, who confirmed the assigned labels against the visible tablet-quality characteristics in each image. Because borderline tablets were removed during quality screening, the reported performance should be read as applying to unambiguous cases; performance on visually marginal tablets, which are a recognized difficulty in manual inspection, is not characterized by this benchmark.

#### 2.1.5. Training, Validation, and Test Splits

The PV dataset is released with fixed training, validation, and test partitions to support consistent and reproducible evaluation across studies. The dataset contains 640 images in total, and its composition is given in Table 1, and the class distribution per partition is given in Table 3.

The training set was used to optimize model parameters during learning; the validation set was used during model development for early stopping and checkpoint selection; and the test set was retained exclusively for final performance evaluation. All three subsets are class balanced, which reduces the risk that classification performance is dominated by the majority class and allows equal emphasis on correct recognition of normal and defective tablets. Balanced testing is particularly useful for evaluating sensitivity to defective tablets, since recall of the defective class directly reflects the proportion of defective images correctly identified.

Partitioning was performed at the level of the source tablet rather than by randomly distributing individual images: all images originating from a given physical tablet, or captured within a single acquisition sequence, were assigned to exactly one partition, so that no physical tablet contributes images to more than one of the training, validation, and test sets. Because the three partitions were additionally acquired in separate sessions approximately two weeks apart (Section 2.1.3), this design prevents two related forms of leakage: the same tablet or defect instance appearing in both training and evaluation, and session-specific acquisition conditions being shared across splits. One consequence of the session separation is that the test set differs from the training set not only in tablet identity but also in acquisition session, so in-domain test performance already incorporates a degree of session-level distribution shift and should be interpreted accordingly. The decision threshold and checkpoint-selection criterion applied to these partitions are described in Section 2.3.

#### 2.1.6. Data Augmentation

**Figure 3:**
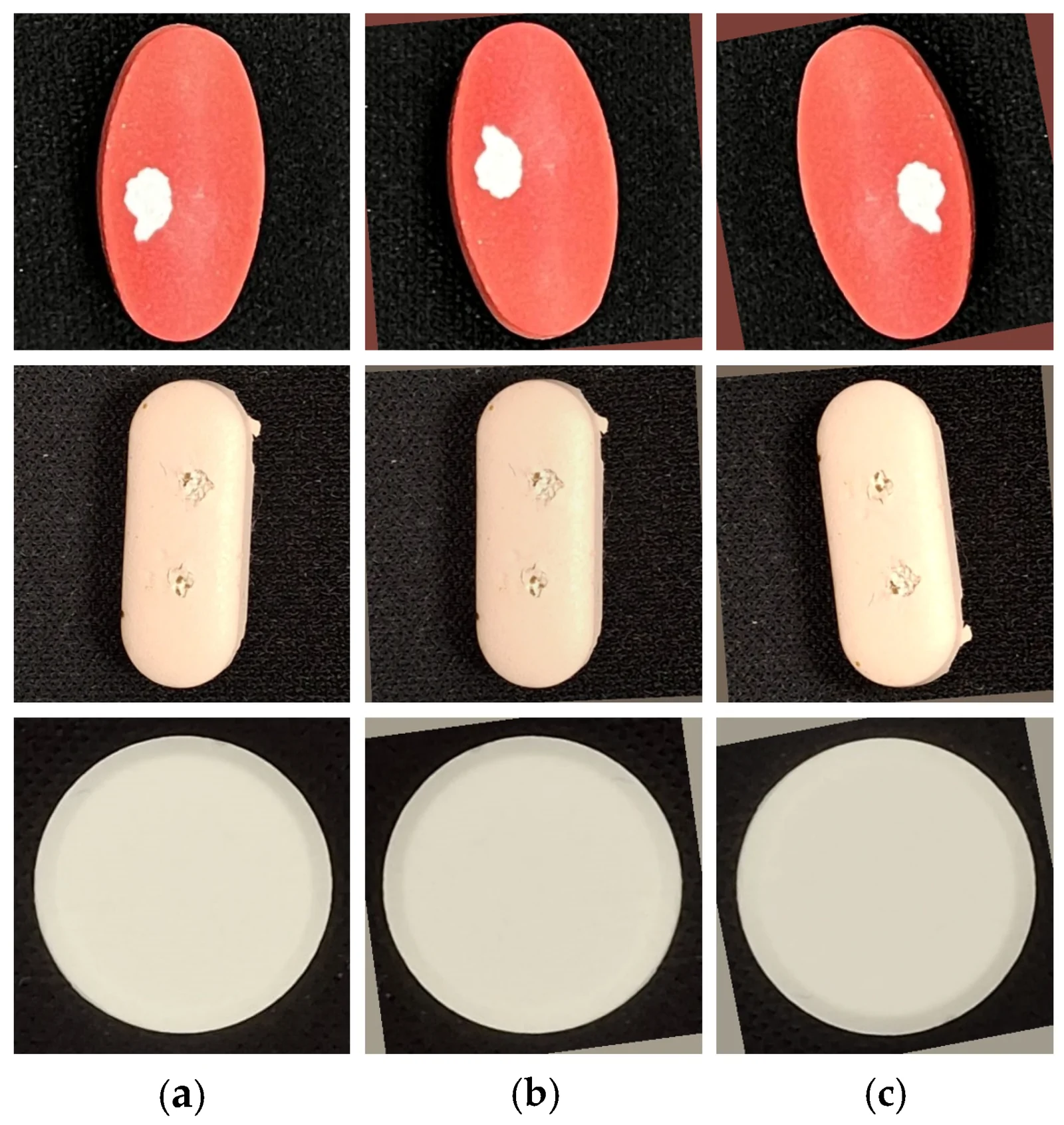
Representative examples of training images before and after offline data augmentation, illustrating the applied flipping, rotation, and brightness/contrast transformations. (**a**) Original images; (**b**) and (**c**) augmented images generated using the applied transformations.

To increase the effective size and visual diversity of the training set, offline data augmentation was applied exclusively to the training partition. Augmentation was performed offline, generating and saving new image files prior to training rather than online during each training iteration, and was never applied to the validation or test sets, so that evaluation was not contaminated with synthetically generated images. For each of the 190 normal and 190 defective training images, two augmented variants were generated in addition to the unmodified original, using mild geometric and illumination transformations: random horizontal and vertical flipping, small-angle rotation (±12°, with the fill color for exposed corners sampled from the source image), and mild brightness and contrast jitter (±7%). The data augmentation expanded the training set from 380 to 1140 images while preserving the original 1:1 class balance, as summarized in Table 4. Figure 3 shows representative examples of original training images alongside their augmented variants.

**Table 4:** Composition of the training set before and after offline data augmentation.

Dataset	Normal Images	Defective Images	Total Images
Original training set	190	190	380
Data augmentation	380	380	760
Total training set	570	570	1140

Augmentation techniques capable of obscuring or fabricating defect evidence, including random cropping, Gaussian blur, Gaussian noise, cutout or random erasing, and hue or saturation shifting, were deliberately excluded from this pipeline. Tablet defects in this dataset are frequently small in spatial extent (e.g., edge chips or hairline cracks) or color-dependent (e.g., staining or discoloration), and such transformations risk removing or distorting the very visual evidence the classifier is trained to detect. Horizontal and vertical flipping were retained despite producing mirror-reversed imprints, which do not occur on physically manufactured tablets, because tablet orientation on an inspection surface is arbitrary and the defects of interest are themselves orientation-independent; the transformation therefore preserves defect evidence exactly while discouraging reliance on imprint content as a class cue. Because flipping was applied equally to normal and defective images, mirror-reversed imprints occur in both classes and cannot act as a class-specific shortcut; future augmentation protocols for imprint-heavy datasets may nevertheless restrict flipping to non-imprinted tablets or use imprint-aware augmentation. The augmented training set is released publicly alongside the original PV dataset.

#### 2.1.7. Cross-Dataset Evaluation on PillQC Dataset

To assess whether the visual representations learned from the PV dataset generalize beyond its source distribution, each model trained exclusively on the PV training set was evaluated, without any fine-tuning or adaptation, on the independent pillQC dataset [21]. Model weights, the classification head, and the decision threshold were held fixed, and pillQC images were passed through each trained model to obtain predictions. Because pillQC does not define an official evaluation split, the full set of images in the evaluated classes was used. Evaluation was restricted to the chip and normal classes, comprising 43 chips and 149 normal images. The dirt class was excluded because it represents deposited foreign particulate on the tablet surface, an externally introduced contamination rather than a structural or manufacturing defect of the tablet itself. The defective class in the PV dataset consists entirely of intrinsic structural, surface, coating, and appearance abnormalities. Including dirt as a positive class would therefore ask models to generalize across a qualitative change in what “defective” means, confounding a domain shift in appearance with a shift in the definition of the target label. For this reason, the evaluation was restricted to the chip and normal classes, where the defect concept is consistent with the PV training objective. Because the dirt class was excluded on conceptual grounds, a sensitivity analysis including dirt would conflate two different definitions of the positive class within the same evaluation and was therefore not performed.

**Figure 4:**
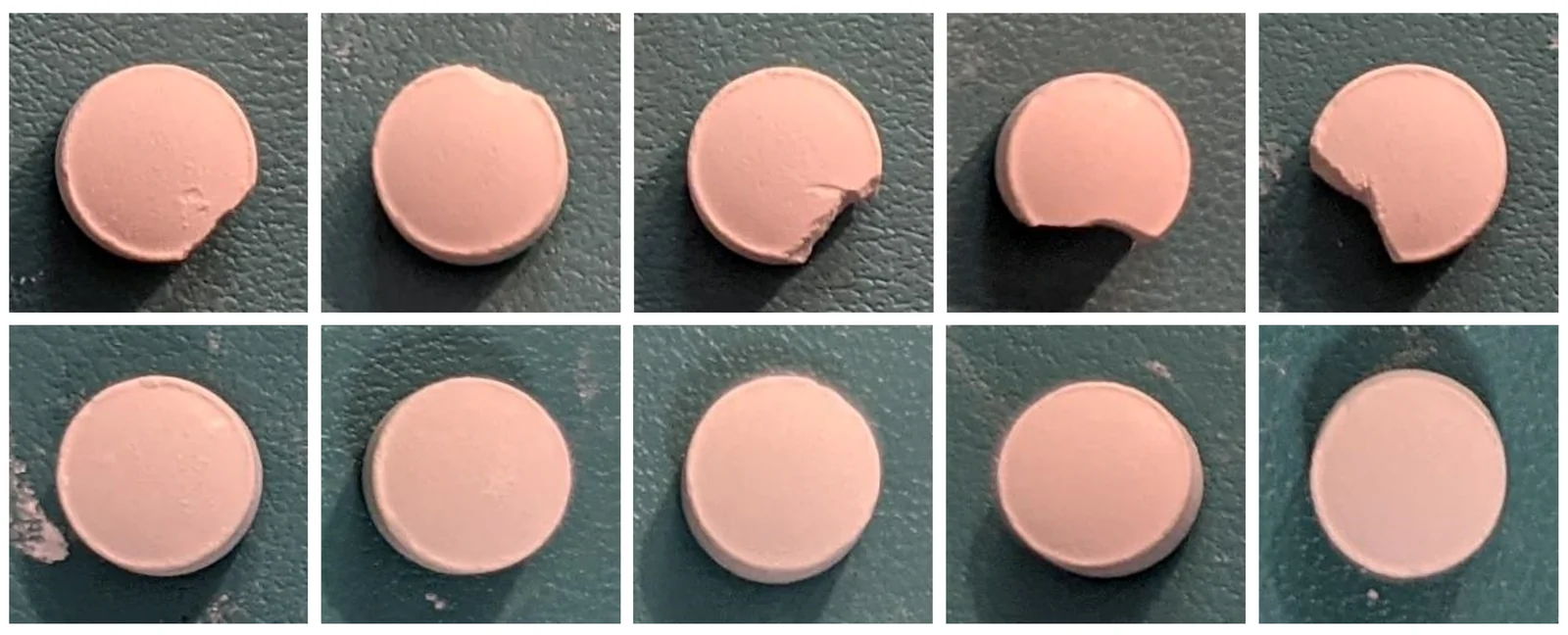
Representative samples from the pillQC used for cross-dataset evaluation, with tablets exhibiting chip defects shown in the top row and normal tablets shown in the bottom row.

This zero-adaptation protocol simulates the scenario in which a model trained on one set of tablet products and imaging conditions is deployed directly against a tablet product, camera setup, and defect appearance it has not encountered during training, providing a stricter test of generalization than in-domain evaluation on the PV test set alone. Two properties of this protocol should be noted when interpreting the results. First, the class balance differs from the PV test set (22.4% positive versus 50%), and because precision and F1-score depend on prevalence, part of any change in these metrics between the two datasets reflects the base-rate difference rather than a change in discriminative ability. Second, the decision threshold was held at its PV-derived value rather than recalibrated for pillQC, so the results characterize zero-adaptation transfer and do not represent an upper bound on achievable cross-dataset performance. Figure 4 shows representative images from the pillQC dataset, and Table 5 presents a structured comparison between the PV and pillQC datasets.

### 2.2. Benchmark Models

State-of-the-art deep learning architectures, spanning classical convolutional networks, computationally efficient and mobile-oriented networks, and vision transformers, were benchmarked on the PV dataset under an identical training and evaluation protocol. Using the same data splits, preprocessing, and training settings for every model ensures that differences in performance can be attributed to the architectures themselves rather than to variations in the training procedure. These architectures range from 1.3 to 140 million parameters and represent three broad design families: convolutional networks relying on stacked or residual convolutions, networks optimized for low parameter count and computational cost, and transformer-based networks that replace convolution with self-attention. This choice is relevant to tablet inspection, because defects such as hairline cracks and edge chips are small, localized features that convolutional networks capture through local receptive fields, whereas transformers model relationships across the entire tablet surface. Mobile-oriented networks were included because a practical inspection system may need to run on smartphones or embedded devices at the point of inspection, consistent with the smartphone-based acquisition used to build the PV dataset. This range allows the effect of architectural family, depth, and parameter budget on tablet defect classification to be assessed directly, alongside the accuracy–efficiency trade-offs reported in Section 3. Together, these results provide a reference baseline against which future methods developed on the PV dataset can be compared. Table 6 summarizes the key design principle of each architecture, and the rationale for its inclusion in the benchmark.

### 2.3. Experimental Setup

All architectures were initialized with ImageNet pretrained weights [34] loaded via torchvision.models using the default weights entry for each architecture, except Xception and DLA-34, which are not available in torchvision and were loaded from the timm library. All layers of every network were fine-tuned, with no layers frozen during training. All architectures shared a single preprocessing pipeline: input images were resized to 224 × 224 pixels and normalized with ImageNet channel means (0.485, 0.456, 0.406) and standard deviations (0.229, 0.224, 0.225). A single input resolution was used for every architecture so that accuracy and computational cost are compared on common ground; this differs from the native evaluation resolutions of some architectures, for example 299 × 299 for Xception and 384 × 384 for EfficientNetV2-S, and may therefore understate their achievable accuracy. Models were trained using the Adam optimizer [35,36,37] with a learning rate of 1 × $10^{-4}$ and a weight decay of 1 × $10^{-4}$ and a batch size of 16, for a maximum of 100 epochs. The learning rate followed a linear warm-up during the first epoch, then cosine decay to 1% of the base value over the remaining schedule. The loss function was standard cross-entropy (PyTorch nn.CrossEntropyLoss with default parameters: no class weighting, no label smoothing, no logit modification), applied identically to every architecture. At inference, a softmax was applied to the two output logits to obtain class probabilities.

Model checkpoints were selected based on the best validation F2-score [38], rather than accuracy or F1-score, computed at a decision threshold of 0.40, selected empirically by evaluating F2-score across candidate thresholds of {0.30, 0.35, 0.40, 0.45, 0.50} on the validation set and choosing the value that maximized the mean validation F2-score across all twelve architectures; this threshold was fixed before any test-set evaluation and applied identically to every architecture. This same 0.40 threshold was applied consistently at test time, for both the in-domain PV test set and the cross-dataset evaluation on pillQC. Training was stopped early if validation F2-score did not improve for 15 consecutive epochs.

The choice of F2-score and a sub-0.50 decision threshold reflects the asymmetric cost structure of pharmaceutical tablet quality control. A false negative, meaning a defective tablet classified as normal, allows a potentially non-conforming product to pass through the inspection stage undetected, with direct consequences for patient safety and regulatory compliance. A false positive, meaning a normal tablet flagged as defective, results in unnecessary review or rejection, which carries an economic cost but does not endanger patients. The F2-score weights recall twice as heavily as precision, penalizing false negatives more heavily than false positives, and a threshold below 0.50 shifts the classifier’s operating point toward higher recall at the cost of some precision, consistent with this priority. Both choices therefore reflect the same underlying judgment about the relative consequences of the two error types in a pharmaceutical inspection context.

The training set was expanded using offline data augmentation prior to training, as described in Section 2.1.6; no augmentation was applied to validation or test images beyond resizing and normalization. The identical training configuration (optimizer, loss function, preprocessing, and augmentation) was used for every benchmarked architecture, with no per-architecture adjustments. Each architecture was trained once, with a fixed random seed of 7 applied to weight initialization of the classification layer, minibatch ordering, and data loading; PyTorch’s non-deterministic CUDA kernels were not disabled, so exact bit-for-bit reproducibility requires the same hardware and software environment. The seed, training code, and data partitions are released with the dataset to allow the experiments to be reproduced. All experiments used PyTorch 2.1.0 [39], torchvision 0.16.0, Python 3.10, and CUDA 12.1 and executed on a workstation with an Intel Core i7-7700 CPU (Intel Corporation, Santa Clara, CA, USA), 24 GB of RAM, and an NVIDIA GeForce RTX 3060 Graphics Processing Unit (GPU) [40] with 12 GB of memory.

### 2.4. Evaluation Metrics

Model performance on the PV test set was assessed using accuracy, precision, recall, and F1-score, with the defective class treated as the positive class, consistent with Section 2.1. For a confusion matrix in which TP, TN, FP, and FN denote true positives, true negatives, false positives, and false negatives with respect to the defective class, the metrics are defined as Equations (1)–(4):

(1) $$\mathrm{Accuracy}=\frac{\mathrm{TP}+\mathrm{TN}}{\mathrm{TP}+\mathrm{TN}+\mathrm{FP}+\mathrm{FN}}$$

(2) $$\mathrm{Precision}=\frac{\mathrm{TP}}{\mathrm{TP}+\mathrm{FP}}$$

(3) $$\mathrm{Recall}=\frac{\mathrm{TP}}{\mathrm{TP}+\mathrm{FN}}$$

(4) $$F1\text{-}\mathrm{score}=\frac{2\mathrm{TP}}{2\mathrm{TP}+\mathrm{FP}+\mathrm{FN}}$$

Recall on the defective class is of practical importance in this setting, since a false negative corresponds to a defective tablet incorrectly classified as normal, the error a quality control screening system is specifically intended to prevent. Because the test sets are small, all metrics are additionally reported with 95% confidence intervals, and differences between architectures are assessed with paired statistical tests (Section 3.3).

In addition to predictive performance, each architecture was profiled for computational efficiency using four measures relevant to deployment in a line-speed inspection setting. Parameters report the total number of trainable parameters in the network, in millions (M), since every layer was fine-tuned (Section 2.3), indicating overall model size and memory footprint. GFLOPs reports the number of Giga floating-point operations required for a single forward pass at a 224 × 224 input resolution, indicating computational cost independent of hardware. Inference latency reports the median wall-clock time, in milliseconds (ms), to process a single image (batch size 1), measured over 500 timed forward passes following 50 warm-up passes, with GPU execution synchronized around each pass; variability is reported as the interquartile range (IQR). Throughput, in frames per second (FPS), is the reciprocal of the median latency and represents a theoretical single-image processing rate, not the end-to-end throughput of an inspection line. Throughput (frames per second) reports the reciprocal of this single-image latency, indicating the maximum image rate an architecture can sustain and directly indicating whether it can keep pace with a production inspection line.

### 2.5. Generative AI Use

During the preparation of this manuscript, Claude Sonnet 5 was used to assist with language editing, sentence restructuring, and improvement of English expression. All AI-assisted output was reviewed and edited by the authors, who take full responsibility for the final content.

## 3. Results

### 3.1. In-Domain Classification Performance on the PV Test Set

Table 7 reports accuracy, precision, recall, and F1-score for all benchmarked architectures on the held-out PV test set, with the defective class treated as positive.

EfficientNetV2-S achieved the highest F1-score (88.56%) and accuracy (88.50%), with balanced precision (88.12%) and recall (89.00%), followed by Xception (87.08%) and Swin-B (85.71%). These differences are, however, within sampling uncertainty: the confidence intervals of the leading architectures overlap, and paired tests showed no significant difference between them (Section 3.3). The more robust observation is that a group of architectures, EfficientNetV2-S, Xception, Swin-B, ShuffleNetV2, and VGG-19, clustered between 84% and 89% F1-score, separated from the weakest performers. AlexNet recorded the lowest accuracy, precision, and F1-score in the benchmark (67.91% F1), and MobileNetV3-small the second lowest (71.28% F1), indicating that both the earliest and the most parameter-constrained architectures struggled comparatively with this task.

Precision and recall did not always move together. ShuffleNetV2 had high precision (92.86%, similar to GoogLeNet and Swin-B) but a comparatively modest recall (78.00%), indicating a conservative decision profile that under-flags defective tablets in exchange for rarely misclassifying normal tablets. Xception showed the opposite pattern, with high recall (91.00%) alongside solid precision (83.49%). Because missed defective tablets are the more consequential error in a quality control setting, a recall-oriented profile of this kind is preferable to a precision-oriented one. EfficientNetV2-S showed a balanced profile, although its recall did not differ materially from that of Xception (89 versus 91 of 100 defective tablets detected).

### 3.2. Cross-Dataset Generalization to the pillQC Dataset

To assess generalization beyond the PV dataset’s source distribution, each model trained exclusively on the PV training set was evaluated, without fine-tuning or adaptation, on the independent pillQC dataset. Results are reported in Table 8. GoogLeNet achieved the highest cross-dataset F1-score (76.19%), followed by EfficientNetV2-S (75.00%), despite its mid-table in-domain performance (83.06%), so the in-domain and cross-dataset orderings did not coincide. The two architectures differ by only two correctly classified images and are statistically indistinguishable (Section 3.3); the substantive finding is the contrast between this leading pair and the vision transformers at the bottom of the table, which is statistically significant.

Every architecture’s F1-score declined from the PV test set to pillQC, but the magnitude of the decline varied substantially. GoogLeNet showed the smallest absolute drop (6.87 percentage points), whereas Swin-B (45.09 points) and ViT (42.93 points) showed the largest, alongside AlexNet (38.85 points) and ShuffleNetV2 (35.34 points). Because each decline is the difference in two estimates with wide confidence intervals, only the large declines should be interpreted. Part of the decline common to all architectures is attributable to the difference in class prevalence between the two evaluation sets (22.4% positive on pillQC versus 50% on the PV test set), since precision and F1-score depend on the base rate; the differences between architectures, which are the basis of the comparisons drawn here, are not affected by this, as all models were evaluated on the same images. Both vision transformers were therefore among the least robust to the distribution shift represented by pillQC, despite Swin-B performing comparably to the leading group in-domain. One possible explanation is the greater data requirement typically associated with self-attention architectures, although this study was not designed to test it (Section 4.1).

A second pattern evident in Table 8 is a systematic drop in recall under distribution shift, larger than the corresponding drop in precision. ResNet-50, for example, retained high precision (96.15%) on pillQC but recall fell to 58.14%; MobileNetV3-small and DenseNet-169 showed an even more pronounced version of this pattern, with recall falling to 30.23% and 34.88%, respectively, while precision remained comparatively high. Recall is unaffected by the prevalence difference between the two datasets, so this asymmetry reflects a genuine change in model behavior rather than a base-rate artifact. In a quality control context, this is the least favorable direction for performance to degrade: under distribution shift, these models became more conservative and missed a larger proportion of defective tablets, rather than failing symmetrically across both classes.

**Table 8:** Cross-dataset classification performance (train: PV training set; test: pillQC without fine-tuning). All values are percentages; bold values indicate the best result in each column. 95% confidence intervals and paired statistical comparisons are reported in Section 3.3.

Method	Accuracy	Precision	Recall	F1-Score
AlexNet [22]	56.77	22.97	39.53	29.06
VGG-19 [23]	88.02	73.81	72.09	72.94
GoogLeNet [24]	89.58	78.05	**74.42**	**76.19**
ResNet-50 [25]	90.10	**96.15**	58.14	72.46
DenseNet-169 [26]	83.33	78.95	34.88	48.39
Xception [27]	88.54	83.87	60.47	70.27
DLA-34 [28]	86.46	79.31	53.49	63.89
ShuffleNetV2 [29]	76.56	47.83	51.16	49.44
MobileNetV3-small [30]	83.33	86.67	30.23	44.83
EfficientNetV2-S [31]	**90.62**	93.10	62.79	75.00
ViT-B/32 [32]	54.17	27.72	65.12	38.89
Swin-B [33]	80.21	61.90	30.23	40.62

### 3.3. Statistical Analysis

The PV test set contains 200 images (100 defective), so a difference of one percentage point in accuracy corresponds to two images, and the pillQC evaluation contains only 43 defective images, so a single image changes recall by 2.33 percentage points. The uncertainty of the results in Table 7 and Table 8 was therefore quantified in two ways: 95% confidence intervals for each architecture (Section 3.3.1) and paired comparisons between architectures (Section 3.3.2).

#### 3.3.1. 95% Confidence Intervals

Table 9 reports the accuracy, precision, recall, and F1-score of each architecture on both test sets together with their 95% confidence intervals. Accuracy, precision, and recall are proportions, and their intervals were computed with the Wilson score method [41,42]. F1-score has no closed-form variance, so its interval was obtained by non-parametric bootstrap [43], resampling test images with replacement 10,000 times and taking the 2.5th and 97.5th percentiles of the resulting F1-scores. On the PV test set, accuracy intervals span approximately span ±5 percentage points, and precision and recall intervals span approximately ±6–9 points. The F1-score intervals of the leading architectures overlap substantially: EfficientNetV2-S (88.56%, 95% CI 83.6–92.9%), Xception (87.08%, 81.9–91.6%), Swin-B (85.71%, 79.8–90.7%), ShuffleNetV2 (84.78%, 78.8–89.9%), and VGG-19 (84.51%, 78.9–89.5%). Their ordering in Table 7 is therefore not statistically established. In contrast, AlexNet (67.91%, 60.4–74.7%) and MobileNetV3-small (71.28%, 63.4–78.3%) lie clearly below this group. On pillQC, the intervals are wider, with most F1-score intervals spanning about 20–30 percentage points. GoogLeNet (76.19%, 64.9–85.4%) and EfficientNetV2-S (75.00%, 62.5–85.1%) overlap almost entirely, and only large differences are interpretable: the F1-score intervals of ViT-B/32 (28.6–48.8%) and Swin-B (24.6–54.8%) do not overlap those of either leading architecture.

#### 3.3.2. Pairwise Comparisons

Overlap between per-model intervals is conservative and does not by itself test whether two models differ. Because all architectures were evaluated on the same images, differences were tested with paired methods: the exact McNemar test [44,45] on per-image correctness, and a paired bootstrap confidence interval for the difference in F1-score, in which the same resampled images were used for both models in each of 10,000 replicates. Comparisons were made between the highest-scoring architecture on each test set and its closest competitors and the weakest architectures, and *p*-values were adjusted with the Holm method [46] across all comparisons in Table 10.

On the PV test set, EfficientNetV2-S did not differ significantly from Xception (ΔF1 = +1.48 percentage points, 95% CI −3.4 to 6.4), Swin-B (+2.85, −2.4 to 8.4), or ShuffleNetV2 (+3.78, −1.7 to 9.6) (McNemar *p* = 0.47–0.57; Holm-adjusted *p* = 1.00 for all three). EfficientNetV2-S classified 177 of 200 images correctly, compared with 173 for both Xception and Swin-B; a four-image difference cannot reach significance under the exact McNemar test however the errors are distributed (minimum attainable *p* = 0.125). Its advantage over AlexNet was significant (+20.65, 13.8 to 28.1; Holm-adjusted *p* < 0.001). On pillQC, GoogLeNet and EfficientNetV2-S differed by only two correctly classified images (172 versus 174; minimum attainable McNemar *p* = 0.50), and GoogLeNet did not differ significantly from VGG-19 (+3.25, −7.1 to 14.0; Holm-adjusted *p* = 1.00). In contrast, GoogLeNet significantly outperformed Swin-B (+35.57, 20.6 to 51.5) and ViT-B/32 (+37.30, 27.7 to 47.4) (Holm-adjusted *p* < 0.001 for both). The benchmark therefore separates a leading group of architectures from the weakest ones and confirms the weaker cross-dataset transfer of the vision transformers, but it does not establish an ordering within the leading group.

**Table 10:** Paired comparisons between architectures evaluated on identical images. ΔF1: difference in F1-score (A, B, percentage points) with 95% paired bootstrap confidence interval (10,000 resamples, identical images drawn for both models); *p*: exact McNemar test on per-image correctness; *p*_Holm: Holm-adjusted *p* across the comparisons in this table.

Test Set	Model A vs. Model B	Correct (A/B)	ΔF1 (95% CI)	*p*	*p*_Holm
PV	EfficientNetV2-S vs. Xception	177/173	+1.48 (−3.4 to 6.4)	0.57	1.00
PV	EfficientNetV2-S vs. Swin-B	177/173	+2.85 (−2.4 to 8.4)	0.57	1.00
PV	EfficientNetV2-S vs. ShuffleNetV2	177/172	+3.78 (−1.7 to 9.6)	0.47	1.00
PV	EfficientNetV2-S vs. AlexNet	177/131	+20.65 (13.8 to 28.1)	<0.001	<0.001
pillQC	GoogLeNet vs. EfficientNetV2-S	172/174	+1.19 (n/a) ^a^	≥0.50 ^a^	1.00
pillQC	GoogLeNet vs. VGG-19	172/169	+3.25 (−7.1 to 14.0)	0.61	1.00
pillQC	GoogLeNet vs. Swin-B	172/154	+35.57 (20.6 to 51.5)	<0.001	<0.001
pillQC	GoogLeNet vs. ViT-B/32	172/104	+37.30 (27.7 to 47.4)	<0.001	<0.001

^a^ Per-image predictions for this evaluation were not retained, so the paired bootstrap interval could not be computed. Because the two models differ by two correctly classified images, the exact McNemar *p*-value cannot be lower than 0.50; however, the errors are distributed.

### 3.4. Model Complexity and Computational Efficiency

Table 11 reports parameter count, GFLOPs, inference latency, and throughput for each architecture. Latency is reported as median and interquartile range (IQR) over 500 inference runs on a single image following 50 warm-up runs; median is used in preference to mean because several architectures exhibited right-skewed latency distributions attributable to GPU scheduling jitter. The reported throughput values represent the theoretical single-image processing rate of each model under the measured conditions and should not be interpreted as the end-to-end throughput of an industrial inspection system, which would additionally depend on image acquisition, preprocessing, data transfer, and pipeline scheduling overhead. AlexNet achieved the highest throughput (602.41 FPS) and lowest median latency (1.66 ms) of any architecture, despite having the fourth-highest parameter count (57.01 M) in the benchmark; its shallow depth of only five convolutional layers, rather than its parameter count, is the more direct explanation for this result. ShuffleNetV2 (1.26 M) and MobileNetV3-small (1.52 M) had the smallest parameter counts and lowest GFLOPs, consistent with their design as mobile-oriented architectures, and both sustained moderate throughput (79.75 and 107.99 FPS respectively). Swin-B recorded the highest median latency (41.19 ms) and lowest throughput (24.28 FPS) of all architectures, despite having fewer parameters than VGG-19 (86.74 M versus 139.58 M); VGG-19, in contrast, achieved higher throughput (146.84 FPS) than its parameter count alone would suggest.

Parameter count and GFLOPs were, on the whole, unreliable predictors of measured latency. DenseNet-169 (12.48 M parameters, 6.87 GFLOPs) and EfficientNetV2-S (20.18 M parameters, 5.80 GFLOPs) both recorded median latencies (40.23 ms and 33.68 ms respectively) far higher than architectures with comparable or larger theoretical compute cost, such as Xception (20.81 M parameters, 9.19 GFLOPs, 11.86 ms) or ResNet-50 (23.51 M parameters, 8.26 GFLOPs, 12.46 ms). These two cases reflect different mechanisms with a common consequence: DenseNet-169’s repeated feature concatenation across densely connected layers, and EfficientNetV2-S’s heavily block-structured design with squeeze-and-excitation operations, are both inexpensive in floating-point operations but do not parallelize as efficiently on GPU hardware as simpler sequential convolutions. GFLOPs and parameter count therefore describe theoretical computational cost, but neither is a substitute for directly measured latency when evaluating deployability on specific hardware.

**Table 11:** Model complexity and inference efficiency. Bold values indicate the best result in each column (lowest parameter count, GFLOPs, and latency; highest throughput). Latency is median (IQR) over 500 single-image inference runs following 50 warm-up runs on an NVIDIA GeForce RTX 3060 GPU. Throughput is a theoretical single-image processing rate (1000/median latency ms) and does not represent end-to-end industrial pipeline throughput (M: million; GFLOPs: giga floating-point operations; ms: milliseconds; FPS: frames per second).

Method	Parameters (M)	GFLOPs	Median Latency (ms)	IQR (ms)	Throughput (FPS)
AlexNet [22]	57.01	1.42	**1.66**	**0.23**	**602.41**
VGG-19 [23]	139.58	39.26	6.81	0.35	146.84
GoogLeNet [24]	5.60	3.02	15.35	8.07	65.15
ResNet-50 [25]	23.51	8.26	12.46	2.55	80.26
DenseNet-169 [26]	12.48	6.87	40.23	5.36	24.86
Xception [27]	20.81	9.19	11.86	2.80	84.32
DLA-34 [28]	15.27	6.18	7.99	1.67	125.16
ShuffleNetV2 [29]	**1.26**	0.30	12.54	3.10	79.74
MobileNetV3-small [30]	1.52	**0.12**	9.26	1.90	107.99
EfficientNetV2-S [31]	20.18	5.80	33.68	4.33	29.69
ViT-B/32 [32]	87.46	5.90	10.40	1.05	96.15
Swin-B [33]	86.74	20.44	41.19	2.97	24.28

### 3.5. Summary of Trade-Offs Across Performance, Generalization, and Efficiency

Considered jointly, Table 7, Table 8, Table 9, Table 10 and Table 11 indicate that no single architecture dominates across predictive performance, cross-dataset generalization, and computational efficiency simultaneously. EfficientNetV2-S was in the leading group on both test sets (in-domain F1-score 88.56%; cross-dataset F1-score 75.00%, statistically indistinguishable from GoogLeNet) with a moderate parameter count (20.18 M), although its measured median latency (33.68 ms) was higher than its GFLOPs would suggest. GoogLeNet, despite mid-table in-domain performance, had the highest cross-dataset F1-score (76.19%), comparable to EfficientNetV2-S, and the smallest decline between the two test sets (6.87 points), together with a small parameter count (5.60 M), making it a reasonable candidate where robustness to unseen imaging conditions is prioritized over peak in-domain accuracy. AlexNet offered by far the highest theoretical throughput of any architecture but the weakest predictive performance on both the PV test set and pillQC, illustrating the classic accuracy–efficiency trade-off in its most extreme form within this benchmark. The vision transformer architectures (ViT and Swin-B), while competitive on the in-domain PV test set, showed the largest declines in cross-dataset F1-score, significantly below the leading CNNs on pillQC, and, in the case of Swin-B, the lowest measured throughput of any architecture evaluated, making them the least attractive choice among the twelve for deployment under the conditions represented by this benchmark.

## 4. Discussion

Reliable automated inspection of pharmaceutical tablets requires not only accurate defect classification but also robustness to changes in tablets, imaging conditions, and data distributions. This study evaluated both properties directly, using an in-domain test set of tablets not seen during training, acquired in a separate session, and an independent cross-dataset evaluation on pillQC with no fine-tuning or adaptation. The principal findings are as follows:

### 4.1. Principal Findings

- No single architecture was simultaneously best on predictive performance, cross-dataset generalization, and computational efficiency. EfficientNetV2-S was consistently in the leading group on both test sets, although its margin over Xception and Swin-B in-domain, and its difference from GoogLeNet on pillQC, was within sampling uncertainty (Section 3.3). GoogLeNet showed the smallest in-domain-to-cross-dataset decline despite weaker in-domain accuracy, and AlexNet was far faster than either despite being the weakest predictive performer in the benchmark. Architecture selection for a real deployment therefore depends on which of these three properties is prioritized, rather than on identifying a single best model.
- The substantially lower cross-dataset performance of ViT-B/32 and Swin-B relative to their in-domain PV results may partly reflect the greater data requirements typically associated with self-attention architectures, which lack the locality and translation-invariance priors of convolutional networks and may therefore generalize less well when trained on a small dataset. However, this study was not designed to isolate the effect of dataset size or architectural inductive bias: the two vision transformers also differ from the convolutional architectures in parameter count, pretraining behavior, and receptive field structure, and the cross-dataset shift simultaneously changes tablet appearance, defect type, class prevalence, and image resolution. The observed performance gap is therefore consistent with a data-size explanation but does not establish it as the causal mechanism.
- The cross-dataset performance differences between architectures on pillQC reflect several simultaneous factors that cannot be disentangled from a single zero-adaptation experiment: the shift in tablet appearance and product diversity, the restriction to chip-only defects, the change in class prevalence from 50% to 22.4%, the use of a PV-derived decision threshold not recalibrated for pillQC, and the difference in image resolution.
- Recall degraded more severely than precision under distribution shift for most architectures. In a pharmaceutical quality control context, this is the least favorable direction of failure: a model that becomes more conservative under distribution shift will miss a larger proportion of genuinely defective tablets, which is the error a screening system is specifically intended to prevent. This reinforces the decision, established in Section 2.3, to select model checkpoints using the F2-score rather than accuracy or F1-score.
- Parameter count and GFLOPs were unreliable predictors of measured inference latency. DenseNet-169 and EfficientNetV2-S recorded latencies higher than architectures with comparable or larger theoretical computational cost, reflecting the limited hardware parallelism of densely connected and block-structured operations relative to simpler sequential convolutions. Architecture selection for line-speed deployment should therefore be based on directly measured latency and throughput on representative hardware, not on parameter count or GFLOPs alone.
- The identical training protocol used across all architectures enables direct and fair comparison of performance under a common set of conditions, which is the intended purpose of the benchmark. However, performance under a standardized protocol should not be equated with the maximum achievable performance of each architecture. Several architectures have native input resolutions that differ from the 224 × 224 pixels used here, for example 299 × 299 for Xception and 384 × 384 for EfficientNetV2-S, and some architectures may benefit from architecture-specific learning rate schedules, augmentation strategies, or longer training. The results therefore characterize how each architecture performs under identical conditions, not how well it could perform if individually optimized.
- The reported latency and throughput values are hardware-specific, measured on an NVIDIA RTX 3060 GPU under laboratory conditions, and reflect relative differences between architectures rather than deployment suitability for a specific industrial inspection line, which depends on additional system-level factors such as camera acquisition rate, image transfer latency, and conveyor throughput.

### 4.2. Practical Implications for Deployment

- Where predictive performance is the priority, EfficientNetV2-S and Xception are the best-supported candidates: their in-domain performance was statistically comparable, and both transferred to pillQC far better than Swin-B. Xception’s measured latency was about one-third that of EfficientNetV2-S (11.86 ms, 33.68 ms).
- Where robustness to unseen tablet products, cameras, or imaging conditions is the dominant priority, GoogLeNet’s cross-dataset performance, comparable to EfficientNetV2-S and with the smallest decline from its in-domain result, combined with its comparatively small parameter count (5.60 M), makes it a reasonable candidate despite its more modest in-domain accuracy.
- Where memory footprint is the binding constraint, as on embedded or edge inspection hardware, ShuffleNetV2 (1.26 M) and MobileNetV3-small (1.52 M) offer parameter counts an order of magnitude below most of the benchmark at moderate throughput. Their generalization, however, was among the weakest measured (49.44% and 44.83% cross-dataset F1), so this footprint advantage should not be assumed to carry over to unseen tablet products or imaging conditions. Notably, neither was the fastest architecture on the hardware used here: AlexNet, VGG-19, and DLA-34 all achieved higher throughput, so a small parameter count should not be equated with low latency.
- Consistent with the framing established in Section 1, a system built on these results is intended to support, rather than replace, human judgment in pharmaceutical quality control. Tablets flagged as defective or classified with low confidence would be referred to trained personnel for confirmation, and final batch-disposition and release decisions would remain the responsibility of qualified QA and QC professionals.

### 4.3. Limitations

- The PV dataset, while designed to include realistic diversity, remains modest in scale (640 raw images, 1140 after offline augmentation) relative to datasets used in general-purpose computer-vision benchmarking. Results obtained here may not fully generalize to larger or more diverse tablet product ranges.
- The test sets are small (200 PV images; 43 defective pillQC images), so 95% confidence intervals span ±5 percentage points for accuracy and ±10 points or more for pillQC recall (Table 9). Differences of a few percentage points between architectures are within sampling uncertainty, and a larger, independently acquired test set would be needed to rank the leading architectures.
- Each architecture was trained once with a fixed random seed. The confidence intervals and paired tests in Section 3.3 quantify uncertainty arising from the finite test sets, but not variation across independent training runs caused by random initialization, minibatch ordering and augmentation. Small differences between the leading architectures may therefore partly reflect run-to-run variability and should not be interpreted as a definitive ranking.
- The defective class mixes naturally occurring production defects (92 images, 28.8%) and defects manually introduced by pharmacists (228 images, 71.3%). Manually prepared defects may differ subtly from production defects, and the reported performance does not distinguish between the two provenance types.
- The current release addresses binary normal-versus-defective classification only. Finer-grained distinctions between defect types (e.g., chipping versus cracking versus discoloration) were not evaluated in this benchmark, although the annotation process recorded sufficient information to support such an extension in future work.
- The fixed decision threshold (0.40) was selected on the PV validation set and applied without adjustment to the pillQC evaluation. Because pillQC’s defect population and class balance differ from those of the PV dataset, this fixed threshold may not represent the optimal operating point for pillQC specifically; the cross-dataset results reported here should therefore be interpreted as a test of zero-adaptation transfer, not as an upper bound on achievable cross-dataset performance.
- Partitioning was performed at the level of the physical tablet, and no tablet appears in more than one partition; however, the same product group can occur in more than one partition, and because batch information was not recorded, batch-level overlap cannot be excluded. Models may therefore partly benefit from product-level familiarity, and in-domain performance should be interpreted accordingly.
- The cross-dataset evaluation on pillQC covers chip-type structural defects only and does not characterize generalization to contamination-type defects such as dirt.
- Cross-dataset evaluation was conducted on a single external dataset (pillQC), representing one tablet product, one imaging configuration, and one defect type. Generalization across different manufacturing environments, production sites, or imaging systems cannot be established from a single external evaluation and requires validation on additional independent datasets.
- The reported results represent performance under a common standardized protocol and should not be interpreted as the maximum achievable performance of each architecture. Per-architecture tuning of input resolution, learning rate schedule, or augmentation may yield higher absolute performance for individual models.

### 4.4. Future Work

- Extending the PV dataset with additional tablet products, manufacturing sites, and imaging devices would allow the generalization patterns observed here, particularly the transformer architecture’s weaker cross-dataset performance, to be tested at a larger scale.
- The per-defect-type information retained during annotation could support a multi-class extension of the PV dataset, enabling defect localization or segmentation in addition to binary screening.
- Evaluating whether a small amount of target-domain fine-tuning or threshold recalibration closes the cross-dataset gap observed on pillQC would clarify how much of the observed generalization loss is attributable to zero-adaptation transfer specifically, as opposed to a more fundamental limitation of the learned representations.
- Repeating the training of each architecture with multiple random seeds and reporting the mean ± standard deviation would quantify run-to-run variability and establish whether differences within the leading group are consistent.
- Provenance-stratified evaluation, reporting performance separately for naturally occurring and manually introduced defects, is identified as a priority for a future dataset release.
- Future dataset extensions should record pre-consensus individual labels to enable formal inter-rater agreement measurement.

## 5. Conclusions

This study introduced the PharmaVision (PV) dataset, a publicly available benchmark for binary classification of normal and defective compressed pharmaceutical tablets, together with a reference evaluation of twelve deep learning architectures spanning classical convolutional networks, mobile-oriented networks, and vision transformers. The dataset combines tablets photographed during real pharmaceutical production with defective tablets prepared under controlled conditions, and was constructed with explicit attention to reproducibility: each image was labeled by the acquiring pharmacist and independently reviewed by a second pharmacist, with disagreements resolved by consensus; images were partitioned at the level of the source tablet to prevent leakage between splits; and the partitions were acquired across temporally separated sessions to increase visual diversity and further reduce the risk of shared acquisition conditions.

Among the benchmarked architectures, EfficientNetV2-S was consistently in the leading group, with the highest F1-score on the PV test set (88.56%) and a cross-dataset F1-score on pillQC (75.00%) statistically indistinguishable from the highest (GoogLeNet, 76.19%); its margin over the other leading architectures was, however, within the sampling uncertainty of the test sets. Cross-dataset evaluation revealed substantial and uneven degradation across architectures, with the vision transformer models showing the largest decline despite competitive in-domain results, and GoogLeNet showing the smallest decline despite comparatively modest in-domain accuracy. As cross-dataset evaluation was conducted on a single external dataset representing one tablet product and one imaging configuration, these generalization findings should be interpreted as indicative rather than definitive, and broader validation across manufacturing environments remains a priority for future work. Computational efficiency profiling further showed that parameter count and theoretical computational cost were unreliable predictors of measured inference latency, and that the reported throughput figures represent theoretical single-image processing rates rather than end-to-end industrial pipeline throughput; underscoring the importance of direct hardware benchmarking when selecting a model for deployment.

Taken together, these results indicate that architecture selection for normal-versus-defective tablet classification should not rely on in-domain accuracy or theoretical efficiency metrics alone, but should be informed jointly by predictive performance, demonstrated generalization beyond the training distribution, and measured inference efficiency on representative hardware. By releasing the PV dataset, its associated training and evaluation code, and this reference benchmark publicly, this study aims to support reproducible development and fair comparison of future AI-based pharmaceutical quality control systems, while emphasizing that such systems are intended to support, rather than replace, the judgment of qualified quality-assurance and quality control personnel.

## Figures and Tables

**Table 3:** Distribution of normal and defective tablet images in the PharmaVision dataset.

Dataset Split	Normal Images	Defective Images	Total Images
Training set	190	190	380
Validation set	30	30	60
Test set	100	100	200
Total	320	320	640

**Table 5:** Comparison of the PV and pillQC datasets.

Characteristic	PV Dataset	pillQC Dataset
Images evaluated	200 (test set; 50% defective)	192 (43 chips, 149 normal; 22.4% defective)
Tablet shape	Round, oval, caplet/capsule-shaped, hard capsule, heart-shaped	Round only
Tablet color	White/cream, yellow, orange/peach, red/pink/maroon, brown/olive, gray/beige, blue, green/teal	Pink/salmon
Coating	Film-coated, uncoated, sugar-coated, hard capsule	Uncoated
Surface features	Score lines, embossed logos, alphanumeric imprints (many tablets)	None visible
Product diversity	91 visually distinct product groups	Single product
Defect types evaluated	Chip, crack, broken, abrasion, discoloration/mottling/specks/stains, lamination	Chip only
Image resolution	Variable (≈80% square crops)	225 × 225 pixels (fixed)
Background	Diverse (varied colors and textures)	Uniform dark teal/gray texture
Lighting	Uncontrolled, varying	Fixed, controlled, even
Imaging device	Two consumer smartphones (Samsung Galaxy A35, A05)	Not specified (controlled setup)
Acquisition setting	Real manufacturing + controlled lab	Fixed controlled laboratory setup

**Table 6:** Summary of the benchmarked deep learning models.

Category	Model	Key Design Principle	Rationale for Inclusion
Classical CNN	AlexNet [22]	Shallow stack of five convolutional layers with ReLU activations and dropout	Classical baseline
	VGG-19 [23]	Deep, uniform stack of small 3 × 3 convolutional filters	Deep, parameter-heavy classical baseline
	GoogLeNet [24]	Inception modules applying multiple filter sizes in parallel	Parameter-efficient, multi-branch CNN design
	ResNet-50 [25]	Residual (skip) connections enabling very deep networks	Widely adopted modern CNN backbone
	DenseNet-169 [26]	Dense connectivity with feature reuse across all preceding layers	Assesses the effect of dense feature reuse
	Xception [27]	Depthwise separable convolutions	Computationally efficient, high-capacity CNN
	DLA-34 [28]	Hierarchical and iterative multi-stage feature aggregation	Assesses structured multi-stage aggregation
Mobile network	ShuffleNetV2 [29]	Grouped convolutions with channel shuffle	Low-parameter, low-FLOP architecture for edge deployment
	MobileNetV3-small [30]	Depthwise separable convolutions, squeeze-and-excitation	Modern, deployment-oriented lightweight baseline
Mobile/efficient CNN	EfficientNetV2-S [31]	Compound depth-width-resolution scaling with fused convolutional blocks	State-of-the-art efficient CNN
Vision transformer	ViT-B/32 [32]	Patch-based tokenization with pure self-attention, no convolutions	Tests a non-convolutional, attention-only architecture
	Swin-B [33]	Hierarchical structure with shifted-window self-attention	State-of-the-art hierarchical vision transformer

**Table 7:** Classification performance on the PV test set (train: PV training set; test: PV test set). All values are percentages; best value per column in bold. 95% confidence intervals and paired statistical comparisons are reported in Section 3.3.

Method	Accuracy	Precision	Recall	F1-Score
AlexNet [22]	65.50	63.48	73.00	67.91
VGG-19 [23]	83.50	79.65	90.00	84.51
GoogLeNet [24]	84.50	91.57	76.00	83.06
ResNet-50 [25]	84.00	84.69	83.00	83.84
DenseNet-169 [26]	80.50	87.65	71.00	78.45
Xception [27]	86.50	83.49	**91.00**	87.08
DLA-34 [28]	81.00	81.63	80.00	80.81
ShuffleNetV2 [29]	86.00	**92.86**	78.00	84.78
MobileNetV3-small [30]	73.00	76.14	67.00	71.28
EfficientNetV2-S [31]	**88.50**	88.12	89.00	**88.56**
ViT-B/32 [32]	82.00	82.65	81.00	81.82
Swin-B [33]	86.50	91.01	81.00	85.71

**Table 9:** Performance with 95% confidence intervals on the PV test set and on pillQC. Values are percentages, shown as point estimate (95% confidence interval). Intervals for accuracy, precision, and recall are Wilson score intervals; intervals for F1-score are percentile bootstrap intervals (10,000 resamples of test images).

Method	Accuracy	Precision	Recall	F1-Score
(a) PV test set (*n* = 200; 100 defective)				
AlexNet [22]	65.50 (58.7–71.7)	63.48 (54.4–71.7)	73.00 (63.6–80.7)	67.91 (60.4–74.7)
VGG-19 [23]	83.50 (77.7–88.0)	79.65 (71.3–86.0)	90.00 (82.6–94.5)	84.51 (78.9–89.5)
GoogLeNet [24]	84.50 (78.8–88.9)	91.57 (83.6–95.9)	76.00 (66.8–83.3)	83.06 (76.8–88.5)
ResNet-50 [25]	84.00 (78.3–88.4)	84.69 (76.3–90.5)	83.00 (74.5–89.1)	83.84 (77.8–89.0)
DenseNet-169 [26]	80.50 (74.5–85.4)	87.65 (78.7–93.2)	71.00 (61.5–79.0)	78.45 (71.4–84.6)
Xception [27]	86.50 (81.1–90.6)	83.49 (75.4–89.3)	91.00 (83.8–95.2)	87.08 (81.9–91.6)
DLA-34 [28]	81.00 (75.0–85.8)	81.63 (72.8–88.1)	80.00 (71.1–86.7)	80.81 (74.3–86.4)
ShuffleNetV2 [29]	86.00 (80.5–90.1)	92.86 (85.3–96.7)	78.00 (68.9–85.0)	84.78 (78.8–89.9)
MobileNetV3-small [30]	73.00 (66.5–78.7)	76.14 (66.3–83.8)	67.00 (57.3–75.4)	71.28 (63.4–78.3)
EfficientNetV2-S [31]	88.50 (83.3–92.2)	88.12 (80.4–93.1)	89.00 (81.4–93.7)	88.56 (83.6–92.9)
ViT-B/32 [32]	82.00 (76.1–86.7)	82.65 (74.0–88.9)	81.00 (72.2–87.5)	81.82 (75.5–87.3)
Swin-B [33]	86.50 (81.1–90.6)	91.01 (83.3–95.4)	81.00 (72.2–87.5)	85.71 (79.8–90.7)
(b) pillQC, cross-dataset (*n* = 192; 43 defective)				
AlexNet [22]	56.77 (49.7–63.6)	22.97 (14.9–33.7)	39.53 (26.4–54.4)	29.06 (17.9–40.0)
VGG-19 [23]	88.02 (82.7–91.9)	73.81 (58.9–84.7)	72.09 (57.3–83.3)	72.94 (61.3–82.7)
GoogLeNet [24]	89.58 (84.5–93.2)	78.05 (63.3–88.0)	74.42 (59.8–85.1)	76.19 (64.9–85.4)
ResNet-50 [25]	90.10 (85.1–93.6)	96.15 (81.1–99.3)	58.14 (43.3–71.6)	72.46 (59.4–83.3)
DenseNet-169 [26]	83.33 (77.4–87.9)	78.95 (56.7–91.5)	34.88 (22.4–49.8)	48.39 (31.9–62.9)
Xception [27]	88.54 (83.3–92.3)	83.87 (67.4–92.9)	60.47 (45.6–73.6)	70.27 (57.1–81.2)
DLA-34 [28]	86.46 (80.9–90.6)	79.31 (61.6–90.2)	53.49 (38.9–67.5)	63.89 (50.0–75.9)
ShuffleNetV2 [29]	76.56 (70.1–82.0)	47.83 (34.1–61.9)	51.16 (36.8–65.4)	49.44 (35.9–61.7)
MobileNetV3-small [30]	83.33 (77.4–87.9)	86.67 (62.1–96.3)	30.23 (18.6–45.1)	44.83 (27.6–59.4)
EfficientNetV2-S [31]	90.62 (85.7–94.0)	93.10 (78.0–98.1)	62.79 (47.9–75.6)	75.00 (62.5–85.1)
ViT-B/32 [32]	54.17 (47.1–61.1)	27.72 (19.9–37.1)	65.12 (50.2–77.6)	38.89 (28.6–48.8)
Swin-B [33]	80.21 (74.0–85.2)	61.90 (40.9–79.2)	30.23 (18.6–45.1)	40.62 (24.6–54.8)

## Data Availability

The PharmaVision (PV) dataset (version 1.0) and the complete training and evaluation code used to produce the results reported in this study are publicly available at https://github.com/tahirlee/pharmaceutical-tablet-defect-detection (accessed on 6 October 2026), and the dataset is also archived on Zenodo https://zenodo.org/records/22773411 (accessed on 6 October 2026), DOI:10.5281/zenodo.22773411). The pillQC dataset used for cross-dataset evaluation is publicly available at https://github.com/matlab-deep-learning/pillQC (accessed on 6 October 2026).

## Abbreviations

The following abbreviations are used in this manuscript:

PV	PharmaVision
AI	Artificial Intelligence
CNN	Convolutional Neural Network
ViT	Vision Transformer
DLA	Deep Layer Aggregation
QA	Quality Assurance
QC	Quality Control
GFLOPs	Giga Floating-Point Operations
FPS	Frames Per Second
TP/TN/FP/FN	True Positive/True Negative/False Positive/False Negative
GPU	Graphics Processing Unit
CPU	Central Processing Unit
RAM	Random Access Memory
